# Integrated Transcriptomic and Metabolomic Analysis of Color Changes in Maize Root Systems Treated with Methyl Jasmonate

**DOI:** 10.3390/biology14091124

**Published:** 2025-08-25

**Authors:** Chao Zhang, Lili Zhang, Huan Guo

**Affiliations:** 1 Shaanxi Key Laboratory of Plant Nematology, Bio-Agriculture Institute of Shaanxi, Xi’an 710043, China; 2 Hybrid Rapeseed Research Center of Shaanxi Province, Xianyang 715105, China

**Keywords:** maize, jasmonate, transcriptomic, metabolomic, anthocyanin, flavonoid

## Abstract

(1) Background: As a high-biomass cereal crop, maize provides substantial raw materials for food, animal feed, and processing industries. Plant root systems, vital for nutritional support, are directly vulnerable to diverse stressors that result in developmental abnormalities. Anthocyanins function as essential antioxidants, serving not only as natural pigments, but also playing crucial roles in plant stress resistance. As an essential plant hormone, jasmonic acid (JA) mediates plant stress adaptation and developmental processes, and is frequently employed to stimulate anthocyanin production. (2) Methods: Due to scarce reports on JA functions in maize, we specifically examined JA-triggered developmental regulation and anthocyanin biosynthesis using transcriptomic and metabolomic analysis. (3) Results: Phenotypic analyses revealed that exogenous JA application promoted culm development and intensified pigmentation, while enlarging the areas of stems and primary roots. Combined with phenotypic variations, our integrated transcriptomic and metabolomic analyses of root tissues also indicated that significantly altered metabolites specifically clustered within the flavonoid biosynthesis pathway. Moreover, GO and KEGG enrichment analyses of the associated differentially expressed genes confirmed their participation in this synthetic pathway with high confidence. These findings strongly suggest that methyl jasmonate (MeJA) exposure primarily modulates flavonoid biosynthesis, particularly through regulation of *F3H* and *DFR* gene expression, thereby enhancing flavonoid/anthocyanin accumulation in roots. Additionally, our correlation analysis of transcription factors revealed that *Zm00001d018097 (MYB)*, *Zm00001d029963 (MYB)*, and *Zm00001d000236 (bHLH)* likely participate in regulating the expression of structural genes, thereby promoting the upregulation of functional gene expression. (4) Conclusions: These results provide a robust framework for deciphering the MeJA-mediated regulation of anthocyanin biosynthesis in maize radicles, specifically demonstrating that *Zm00001d018097 (MYB)*, *Zm00001d029963 (MYB)*, and *Zm00001d000236 (bHLH)* coordinately enhance the expression of *F3H* and *DFR*.

## Simple Summary

Maize roots are vulnerable to environmental stress, impacting yield. Anthocyanins, crucial antioxidants aiding stress resistance, can be induced by the plant hormone JA, but its role in maize remains poorly characterized. This study investigated how MeJA regulates root development and anthocyanin biosynthesis. Exogenous MeJA application enhanced stem growth while intensifying pigmentation. Integrated transcriptomic and metabolomic analyses of roots revealed MeJA specifically activates the flavonoid biosynthesis pathway. This occurs primarily through upregulation of key structural genes *F3H* and *DFR*, driven by the coordinated action of transcription factors *Zm00001d018097 (MYB)*, *Zm00001d029963 (MYB)*, and *Zm00001d000236 (bHLH)*. These results provide a framework for understanding JA-mediated stress tolerance through the regulation of anthocyanins in maize.

## 1. Introduction

The accumulation of anthocyanins, a class of flavonoids responsible for the red, purple, and blue colors in plants, and widely existing in the roots, stems, leaves, flowers, fruits and seeds of plants in nature, are a family of water-soluble flavonoids. They can be conducive to improving the abilities of plants to reproduce offspring and resist adverse environments [1]. Plant roots, as primary nutrient acquisition organs, incur direct damage from environmental stressors causing developmental defects. Anthocyanins—functioning as dual-role metabolites—provide not only natural pigmentation, but also essential antioxidant defenses that enhance stress resilience [2,3,4]. The biosynthesis of anthocyanins is regulated by a series of enzymatic reactions and transcriptional factors that control the flow of metabolites through the flavonoid pathway [5]. Anthocyanins are synthesized through a complex pathway involving multiple enzymes, including phenylalanine ammonia-lyase (PAL), chalcone synthase (CHS), chalcone isomerase (CHI), flavanone 3-hydroxylase (F3H), dihydroflavonol 4-reductase (DFR), anthocyanidin synthase (ANS), and UDP- glucose: flavonoid 3-O-glucosyltransferase (UFGT), which is responsible for the final glycosylation step in anthocyanin biosynthesis [6,7,8]. The expression of these structural genes is modulated by a complex network of transcription factors, such as the R2R3-MYB, bHLH, and WD40 proteins, which form the regulatory complex known as the MBW complex [9,10].

Recent investigations have employed transcriptomic and metabolomic strategies to pinpoint key genes and metabolic pathways that regulate anthocyanin biosynthesis. For example, a study on mulberry utilized comparative transcriptome analysis to elucidate the mechanisms behind anthocyanin biosynthesis in black and white fruit genotypes, identifying genes with differential expression during fruit development [11]. Another study on the pagoda tree discovered candidate genes involved in anthocyanin accumulation in the flowers through transcriptome analysis, highlighting the role of transcription factors such as MYBs and bHLHs in the regulation of anthocyanin biosynthesis [12]. Similarly, research on wheat grains during the grain-filling stage combined transcriptomics with metabolomics to identify genes and metabolic pathways related to grain color formation, underscoring the significance of the flavonoid biosynthesis pathway in this process [13]. These studies illustrate the potential of integrating transcriptomic and metabolomic data to clarify the complex regulatory networks that govern anthocyanin production in plants.

Jasmonate, a class of plant hormones, is instrumental in a multitude of biological processes, including plant development, defense against herbivores, regulation of flowering, and adaptation to environmental stresses [14,15]. Methyl Jasmonate (MeJA), a volatile organic compound, has been identified as a critical regulator in the biosynthesis of secondary metabolites, including flavonoids, alkaloids, and terpenoids [16,17]. Furthermore, MeJA serves as a signaling molecule that induces the expression of defense-related genes and enhances the production of secondary metabolites in response to biotic and abiotic stresses [18]. The application of MeJA as an elicitor in plant biotechnology has been demonstrated to increase the accumulation of anthocyanins in various plant species, such as grapes, blackberries, strawberries and apple fruits, thereby improving their coloration and potential health benefits [19,20,21].

Some researchers had utilized transcriptomic and metabolomic techniques to explore a new regulatory gene in the promotion of plant anthocyanin accumulation by MeJA [22]. The researcher indicated that the MYB75 and bHLH factors (GL3 and EGL3) are essential components of WD-repeat/bHLH/MYB transcriptional complexes, to repress JA-regulated anthocyanin accumulation [23]. The study provided an updated perspective on the role of jasmonate in plant growth, development, and the elicitation of secondary metabolites, emphasizing the potential of using exogenous jasmonate to stimulate the production of bioactive compounds in medicinal plants [8]. The research demonstrated that MeJA and the MeJA-induced gene, MdMYB24, is involved in the regulation of anthocyanin and proanthocyanidin biosynthesis in apples [24]. The research by An et al. (2021) revealed that the JA signaling repressor MdJAZ1 interacts with MdTRB1, disrupting its interaction with MdMYB9, and thus negatively modulating the biosynthesis of anthocyanin and proanthocyanidin through transcriptional analysis [25]. These studies underscore the complexity of the jasmonate-responsive transcriptome and its implications for enhancing the nutritional and sensory qualities of fruits.

Transcriptome analysis has emerged as a powerful tool to understand the molecular mechanisms that underlie anthocyanin accumulation. By comparing the transcriptomes of plants treated with MeJA to those of untreated controls, researchers can identify differentially expressed genes that are potentially involved in the regulation of anthocyanin biosynthesis [26]. By identifying key structural and regulatory genes, these approaches can facilitate the development of strategies for improving the nutritional value and aesthetic appeal of plants through breeding or genetic modification.

Building upon this foundation, our work mechanistically interrogates MeJA regulation of anthocyanin dynamics in maize primary roots. Through integrated transcriptome–metabolome profiling, we delineate previously uncharacterized genetic networks and metabolic routes governed by MeJA. These findings resolve critical knowledge gaps in the crosstalk between jasmonate signaling and flavonoid metabolism, ultimately elucidating how root-localized anthocyanin accrual enhances stress resilience in maize.

## 2. Materials and Methods

### 2.1. The Cultivation and Treatment of Plant Materials

We utilized inbred parental lines (≥F6) with homozygous black kernels, green culms, and white root architectures, which the phenotypes stabilized through six generations of enforced self-pollination. Seedlings were planted into 12 pots (of 600 mL) filled with peat–vermiculite substrate (2:1 *v*/*v*), with two uniform seedlings per pot maintained for each biological replicate. Uniformly germinated seedlings were treated 7 days with 50 mL of Hogland nutrient solution (Solarbio, Beijing, China) containing 50 µM MeJA (Solarbio, Beijing, China) per pot, and controls received solution without MeJA (Table S11). Uniform V5-V6 developmental stage (5–6 collared leaves; 30 ± 2 DPE) were selected for phenotypic trait quantification: plant height, root length, stem length, stem diameter, and leaf number, measured using a vernier caliper (0.1 mm, DELI, Hangzhou, Zhejiang, China). Moreover, visual assessment of color differences in primary roots was also conducted. Primary roots were harvested from six biological replicates before and after treatment for transcriptome sequencing (R_0_1, R_0_2, R_0_3, R_0_4, R_100_1, R_100_2, R_100_3 and R_100_4), untargeted metabolomic profiling (whole biological replicates), and anthocyanin compound detection (R_0_1, R_0_2, R_0_3, R_100_1, R_100_2 and R_100_3).

Primary root tissues were dissected from maize seedlings, flash-frozen in liquid nitrogen, and cryogenically ground to fine powder. Samples were precisely weighed to 50 mg and extracted with 0.5 mL methanol/water/hydrochloric acid (500:500:1, *V*/*V*/*V*) for anthocyanin metabolites analysis. Then the extract was vortexed for 10 min and centrifuged at 12,000× *g* under 4 °C, for 3 min. The residue was re-extracted by repeating the above steps again under the same conditions. The supernatants were collected, and filtrated through a membrane filter (0.22 µm, Anpel, Shanghai, China) before LC-MS/MS QTRAP 6500+ (SCIEX, Maryland, DC, USA) analysis.

Phenotypic differences and variations in anthocyanin and flavonoid content were statistically compared using one-way analysis of variance (ANOVA), which was performed using IBM SPSS Statistics 31 (Duncan test, *p* < 0.05), and GraphPad Prism 8.0 software was used for drawing the bar graph.

### 2.2. Library Construction and Sequencing for Transcriptome Analysis

Total RNA was extracted from the samples using the TRIzol Total RNA Extraction Kit (Invitrogen, Carlsbad, CA, USA). Following extraction, the purity and integrity of the RNA were assessed using instruments including 1% agarose gel electrophoresis, and imaged using a Gel Doc™ XR+ System (Bio-Rad, Hercules, CA, USA) under standardized UV illumination. In addition, the Bioanalyzer 2100 system (Agilent Technologies, Santa Clara, CA, USA) was used for analysis of RNA molecular weight determination, quantitative analysis and sample quality control.

Strand-specific libraries were prepared using the NEBNext Ultra Directional RNA Library Prep Kit for Illumina (New England Biolabs, Ipswich Town F.C., Ipswich, MA, USA). mRNA was fragmented into short fragments using fragmentation buffer. Subsequently, first-strand cDNA was synthesized by reverse transcription using random hexamers primed on mRNA templates, followed by second-strand cDNA synthesis with the addition of buffer, dNTPs, and DNA polymerase I. The double-stranded cDNA was then purified using AMPure XP beads (Beckman Coulter, Pasadena, CA, USA). The purified double-stranded cDNA underwent end repair, A-tailing, and adapter ligation. After passing quality control, the libraries were ultimately sequenced on an Illumina NovaSeq 6000 system (Illumina, San Diego, CA, USA) with paired-end 150 bp (PE150) sequencing.

Upon completion of sequencing, raw data in FASTQ format were obtained. To enhance data analysis reliability, Trimmomatic v0.33 was employed to filter out adapter-containing reads, reads with >10% undetermined bases (N), and low-quality reads (where >50% of bases had Q ≤ 20), yielding clean reads for subsequent analysis. The clean reads were mapped to the maize reference genome (B73 RefGen_v4, MaizeGDB) using HISAT2 (v2.2.1). A mapping rate >90% for high-quality reads ensured sufficient data availability for subsequent analyses. Gene-level read counts were subsequently calculated for the aligned reads with HTSeq (v0.13.5). Differential expression analysis was conducted with DESeq2 (v1.38.0) using a negative binomial model, with significance thresholds set at adjusted *p*-value < 0.05 and |log2(fold-change)| > 1. Transcriptomic data were subjected to hierarchical clustering, principal component analysis (PCA), and inter-sample Pearson correlation coefficient calculations using R software v4.2.0. Furthermore, GO and KEGG pathway enrichment analyses of differentially expressed genes (DEGs) were performed with clusterProfiler v4.4.4 and visualized via GOplot package v1.0.2 in R.

### 2.3. Metabolomic Library Construction and Analysis

Metabolite profiling was conducted on twelve biological samples (six biological replicates). Samples were freeze-dried under vacuum and then homogenized to a fine powder using a ball mill (30 Hz, 1.5 min; Retsch, Haan, Germany). Exactly 100 mg of powdered material was extracted in 1.2 mL of 70% (*v*/*v*) methanol. Samples underwent intermittent vortexing (30 s) at 30 min intervals for six cycles, and were then incubated overnight at 4 °C. After centrifugation (12,000× *g*, 10 min, 4 °C), supernatants were collected, filtered through 0.22 μm nylon membranes, and transferred to autosampler vials. Chromatographic separation and mass spectrometric detection were performed using a Nexera X2 UPLC system (Shimadzu Corporation, Kyoto, Japan) coupled to a QTRAP 4500 mass spectrometer (SCIEX, Maryland, DC, USA).

Chromatographic separation was performed on an Agilent SB-C18 column (2.1 × 100 mm, 1.8 µm particle size). The mobile phase consisted of (A) 0.1% (*v*/*v*) formic acid in purified water (Wahaha, Zhejiang, Hangzhou, China) and (B) 0.1% (*v*/*v*) formic acid in acetonitrile. The gradient program was 0–9 min, 5–95% B; 9–10 min, 95% B; 10–11.1 min, 95–5% B; 11.1–14 min, 5% B. The flow rate was 0.35 mL/min, with column temperature maintained at 40℃, and injection volume set to 4 µL.

Mass spectrometric analysis employed electrospray ionization (TurboSpray, SCIEX, Maryland, DC, USA) with source temperature 550 °C and voltages of +5.5 kV (positive mode) or −4.5 kV (negative mode). Source gases were set to GS1: 50 psi, GS2: 60 psi, and curtain gas: 25 psi. Collision-induced dissociation utilized the ‘High’ instrument preset. System calibration was performed with polypropylene glycol solutions (10 μmol/L for QQQ mode; 100 μmol/L for LIT mode). Quantitative MRM scans used nitrogen collision gas (“medium” pressure setting) in QQQ mode. Declustering potential and collision energy were optimized for each metabolite transition. Scheduled MRM scanning was implemented with retention time-specific windows.

### 2.4. Metabolomic Profiling Analysis

Chromatographic peaks were processed in Analyst 1.6.3 and MultiQuant 3.0.3 by alignment with authentic standards’ retention times and fragmentation patterns. Metabolite identification utilized an in-house database (MetWare Database, MWDB: https://cloud.metware.cn/#/data-collection/preview-center (accessed on 2 July 2025)) containing reference MS/MS spectra and retention indices. Multivariate analyses (PCA and OPLS-DA) were performed to identify differential metabolites. Differentially expressed metabolites (DEMs) were defined by |log2(fold-change)| ≥ 0.584 (equivalent to 1.5-fold change), VIP > 1 from OPLS-DA, and *p* < 0.05. Heatmaps visualizing DEMs were created with ComplexHeatmap (v2.14.0) in R (v4.2.0). KEGG pathway enrichment analysis was conducted with clusterProfiler (v4.4.4).

### 2.5. Correlation Analysis

Metabolic-hub gene and transcription-factor structural gene correlations were assessed by Spearman’s rank correlation analysis implemented in R (v4.2.0). For comparison of individual treatments with relevant controls, paired two-tailed Student’s *t*-tests in R (v4.2.0) were performed, and *p*-value ≤ 0.05 was considered statistically significant.

### 2.6. Identification of Real-Time qRT-PCR

For validation of the RNA-seq results, twenty differentially expressed genes (DEGs) were subjected to quantitative real-time PCR (qRT-PCR) (AB-7500, Applied Biosystems, Carlsbad, CA, USA) analysis. cDNA synthesis was performed with Hifair^®^ III 1st Strand cDNA Synthesis SuperMix (gDNA digester plus; Yeasen, Shanghai, China), followed by qPCR amplification using Hieff^®^ qPCR SYBR Green Master Mix (Low Rox Plus; Yeasen, Shanghai, China) for qPCR reaction. The endogenous control gene employed was *Zm00001d046449 (EF-1α)* [27,28]. Gene-specific primer sequences are provided in Table S8. PCR amplification conditions and calculation followed established protocols [29]. The data analysis and drawing also used the method from ‘The cultivation and treatment of plant materials’ (above).

## 3. Results

### 3.1. The Phenotypes and Anthocyanin Content Under the MeJA Treatment

**Figure 1:**
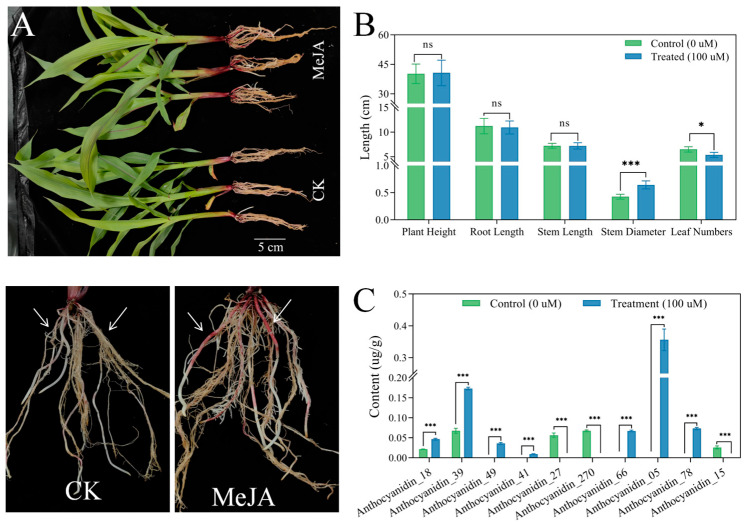
MeJA induced differences in maize development and root anthocyanin accumulation. (**A**) MeJA induced phenotypic changes in maize primary root color (The arrow indicates the difference in color of the primary roots). (**B**) The impact of MeJA induction on maize development (***: *p* value < 0.001; *: *p* value < 0.05; ns: *p* value > 0.05). (**C**) Differences in anthocyanin content in the primer root (fresh samples) under MeJA treatment (***: *p* value < 0.001).

The results of phenotypic statistical analysis of MeJA-treated maize samples and control samples showed that the plant height and stem diameter were reduced under the MeJA treatment, but the stem diameter was wider than the control (Figure 1A). Additionally, when comparing and contrasting the morphologies of roots alongside color alterations, it was determined that the region of color distribution and intensity were influenced by the exposure to the MeJA (Figure 1B). Consequently, our investigation delved into the analysis of anthocyanin content and composition of root under the various MeJA treatments, respectively (Figure 1C). The examination results indicated a significant increase in the overall anthocyanin levels within root upon treatment with MeJA when compared with untreated samples, with Delphinidin, Malvidin-3-O-arabinoside, Quercetin-3-O-glucoside, Peonidin-3,5-O-diglucoside, Naringenin, Pelargonidin-3-O-arabinoside, Peonidin-3,5-O-diglucoside and Cyanidin-3-O-(6-O-malonyl-beta-D-glucoside), exhibiting a notably higher concentration than its counterparts. On the contrary, however, the level of Cyanidin-3-O-sophoroside, Delphinidin-3-O-glucoside and Pelargonidin-3-O-xyloside remained below that of the control group (Table S1).

### 3.2. Transcriptome Analysis of Maize Seedlings Under MeJA Treatments

Our transcriptomic investigation employed mRNA sequencing to analyze eight maize specimens subjected to MeJA treatments using the Illumina NovaSeq platform (San Diego, CA, USA), generating 339,001,508 raw reads (51.19 GB) of primary data. Following stringent quality control, including adapter removal and low-quality read filtration, we retained 331,800,444 clean reads (49.90 GB) with exceptional quality metrics. Sequencing data passed quality control with Q20 ≥ 98.50% (Phred score threshold = 20, error rate < 1%) and GC content = 51.79%, exceeding minimum requirements for bioinformatic analyses. Alignment against the maize B73 reference genome (v4.43) achieved an average mapping rate of 90% (SD), demonstrating high analytical utility for subsequent transcriptional profiling and differential expression analysis (Table S2).

**Figure 2:**
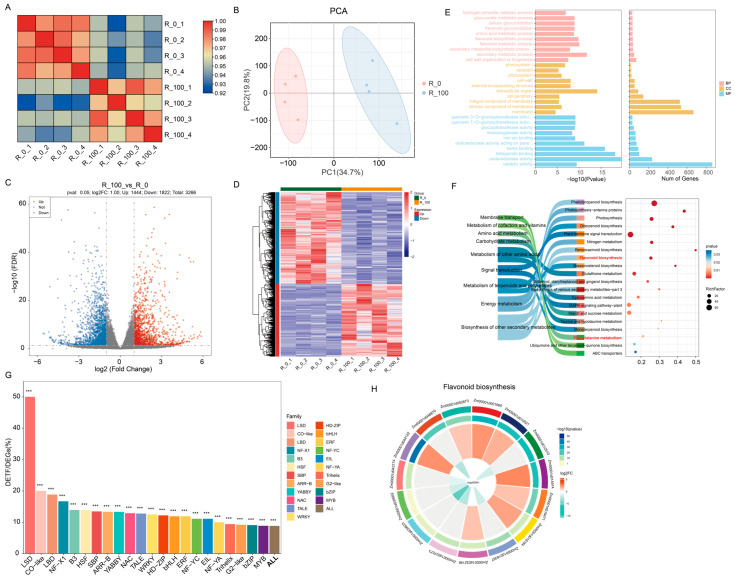
Analysis of differential transcriptional sequencing. (**A**) Analysis of transcriptional differences within treatments; (**B**) PCA analysis of differences between treatments; (**C**) volcano plot of differential gene expression distribution (Blue represents negative regulation; Red indicates positive regulation.); (**D**) Heatmap of clustered differentially expressed genes; (**E**) GO enrichment analysis of differentially expressed genes (BP: Biological Process; MF: Molecular Function; CC: Cellular Component); (**F**) KEGG enrichment analysis of differentially expressed genes; (**G**) statistics of differentially expressed transcription factors (***: *p* value > 0.001 by Fisher test); (**H**) analysis of differentially expressed genes in the flavonoid biosynthesis pathway.

Principal component analysis (PCA) revealed that the transcriptional data between the control group (R_0) and the 100 µM MeJA treatment group (R_100) were markedly different. The correlation plot among samples demonstrated high intra-group reproducibility (Figure 2A). The distinct separation of different treatment groups on the PCA plot indicates significant inter-group differences (Figure 2B). To explore the differentially expressed gene (DEG) in maize roots under MeJA stress, DEseq2 software was used to screen DEGs between R_100 vs. R_0 groups with a threshold of *p* value < 0.05 and |Log2FC| >1. A total of 3266 differentially expressed mRNAs were identified, including 1444 upregulated and 1822 downregulated, as shown in Figure 2C. The heat map of expression distribution of DEGs for each sample is presented in Figure 2D, which shows significant expression differences between R_100 and R_0, with samples clearly divided into two groups.

Gene ontology (GO) enrichment analysis of DEGs showed that the significantly enriched biological processes showed there were three terms directly related to the flavonoid process. They were flavonoid glucuronidation (32 genes), the flavonoid biosynthetic process (36 genes) and the flavonoid metabolic process (37 genes). On the other hand, the secondary metabolite biosynthetic process (37 genes) and the secondary metabolic process (50 genes) also participated in the flavonoid process. The significantly enriched cellular component terms were related to the membrane (an integral component of the membrane (526 genes), the intrinsic component of the membrane (538 genes), and the membrane (669 genes)). In addition, the significantly enriched molecular function terms, quercetin 3-O-glucosyltransferase activity (31 genes), quercetin 7-O-glucosyltransferase activity (31 genes), glucosyltransferase activity (46 genes) and monooxygenase activity (64 genes), were associated with flavonoid regulation or synthesis (Figure 2E and Table S3). The Kyoto Encyclopedia of Genes and Genomes (KEGG) enrichment analysis revealed that the enriched pathways were primarily categorized under Metabolism. The enriched pathways between groups were also enriched in Phenylpropanoid biosynthesis (map00940) with 63 genes (*p* = 1.48637 × 10^−18^), Flavonoid biosynthesis (map00941) with 15 genes, and Phenylalanine metabolism (map00360) with 8 genes (*p* = 0.010449); these could all be related to phenylpropanoid and flavonoid compounds, which was consistent with the GO enrichment results (Figure 2F and Table S3).

In addition, transcription factor analysis of DEGs identified a total of 378 differential TFs (Figure 2G). Significance analysis via Fisher’s exact test for individual transcription factors revealed universally extreme statistical significance (*p* < 0.0001), with the top five in terms of quantity being bHLH (43 genes), ERF (33 genes), NAC (32 genes), MYB-related (29 genes) and bZIP (20 genes). Conversely, the top five transcription factors by proportion of DEGs to total family members were identified as LSD (50%), CO-like (20%), LBD (18.84%), NF-X1 (16.67%) and B3 (13.92%). And these all belong to small-sized TF families in plants.

### 3.3. Analysis and Enrichment of Metabolic Processes Under MeJA Treatments

**Figure 3:**
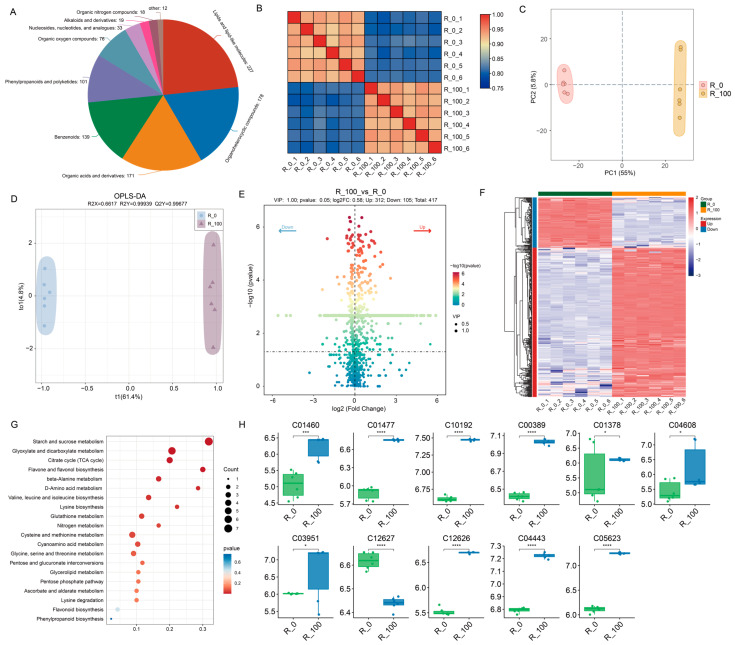
Metabolomics differential analysis. (**A**) Classification and statistics of differential metabolites; (**B**) heatmap analysis of differences among samples within treatments; (**C**) PCA analysis of samples between treatments; (**D**) OPLS-DA analysis of samples between treatments; (**E**) distribution analysis of differential metabolites; (**F**) heatmap cluster analysis of differential metabolites; (**G**) KEGG enrichment analysis of differential metabolites; (**H**) analysis of differences in flavonoid metabolites (****: *p* value < 0.0001, ***: *p* value < 0.001, *: *p* value < 0.05).

To investigate the metabolic differences in maize roots under different stress treatments, UPLC/ESI-QTRAP-MS/MS identified 1296 metabolites, mainly in categories like Lipids and lipid-like molecules (227), Organoheterocyclic compounds (178), Organic acids and derivatives (171), Benzenoids (139), Phenylpropanoids and polyketides (101), Organic oxygen compounds (76), Nucleosides, nucleotides, and analogues (33), Alkaloids and derivatives (19), and Organic nitrogen compounds (18), as shown in Figure 3A. PCA and correlation analysis of metabolites revealed good reproducibility within groups and significant differences between groups, consistent with the transcriptome results in Figure 3B,C. The OPLS-DA model for R_100 vs. R_0 (Figure 3D) had R2X = 0.6617, R2Y = 0.9999, and Q2Y = 0.9967, indicating a well-fitting, reliable model. Using thresholds of |Log2FC| > 0.584, VIP > 1, and *p* < 0.05, 417 DEMs were identified, with 312 upregulated and 105 downregulated (Figure 3E). The heatmap of DEMs divided them into two main groups, with most showing increased levels following induction treatment (Figure 3F).

KEGG enrichment analysis of these DEMs highlighted pathways such as Starch and sucrose metabolism, Citrate cycle (TCA cycle), Flavone and flavonol biosynthesis, Flavonoid biosynthesis, and Phenylpropanoid biosynthesis (Figure 3G and Table S4). Three metabolic pathways were associated with flavonoid synthesis and were also predicted in the transcriptome analysis. Therefore, we extracted 11 differential metabolites from the flavonoid metabolic pathway for comparative analysis to clarify the differences between the control and treated groups. Most metabolites were significantly higher in the treated group than in the control group, except for C12627 (Rhoifolin) and C01378 ((-)-Fustin). The extremely significant differential metabolites were mainly C12627 (Rhoifolin), C01477 (Apigenin), C10192 (Tricetin), C00389 (Quercetin), C12626 (Trifolin), C05623 (Quercetin-3-D-glucoside) and C04443 (3-Methoxy-5,7,3′,4′-tetrahydroxy-flavone) (Figure 3H).

### 3.4. Combined Transcriptomics and Metabolomics Analysis of Maize Response to MeJA-Induced Gene

The integration of metabolome and transcriptome data explored the differential impacts of MeJA-treatment on maize. We subsequently performed KEGG pathway enrichment analysis on the DEGs and DEMs. The enrichment analysis revealed significant enrichment in the Flavonoid biosynthesis and phenylpropanoid biosynthesis pathways under MeJA-treated conditions (Figure 2F and Figure 3G).

**Figure 4:**
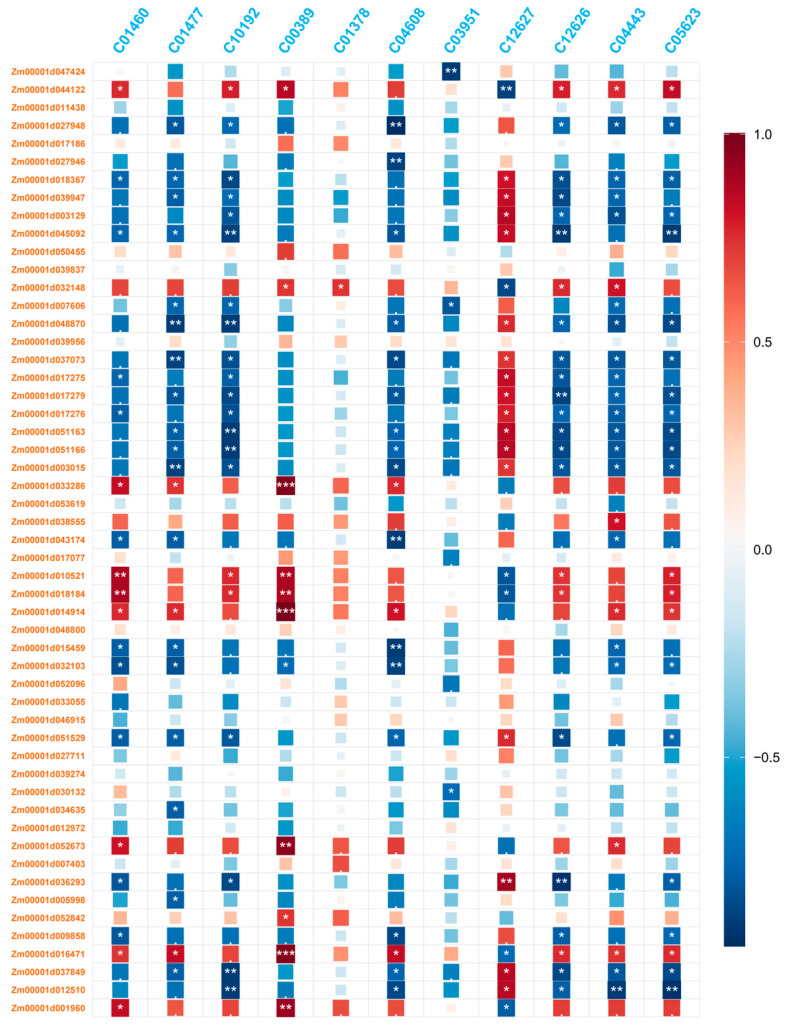
Correlation analysis between differential genes related to flavonoid biosynthesis and metabolites. (***: *p* value < 0.001; **: *p* value < 0.01; *: *p* value < 0.05.)

In analyzing the flavonoid synthesis pathways (map00941 and map00944), we identified 17 structural genes, K10775 (PAL), K01904 (4CL), K00487 (C4H/CYP73A), K00660 (CHS), K01859 (CHI), K00475 (F3H), K05278 (FLS), K09754 (CYP98A, C3’H), K13083 (CYP75A), K13082 (DFR), K05277 (ANS/LDOX), K13081 (LAR), K05280 (CYP75B1), K00588 (COMT), K13065 (HCT), K21102 (ANR), and K13077 (FNSI). These genes corresponded to 54 DEGs in maize (excluding Novel and those with low expression average FPKM > 1) and 11 DEMs (C01460 (vitexin), C01477 (Apigenin), C10192 (Tricetin), C00389 (Quercetin), C01378 ((-)-Fustin), C04608 (Apigetrin), C03951 (Cynaroside), C12627 (Rhoifolin), C12626 (Trifolin), C04443 (3-Methoxy-5,7,3′,4′-tetrahydroxy-flavone), and C05623 (Quercetin-3-D-glucoside)). The heatmap illustrating the correlation between metabolites and hormone-related differentially expressed genes is presented in Figure 4 (Table S5). *Zm00001d033286*, *Zm00001d014914* and *Zm00001d016471* showed positive correlations with C12626.

**Figure 5:**
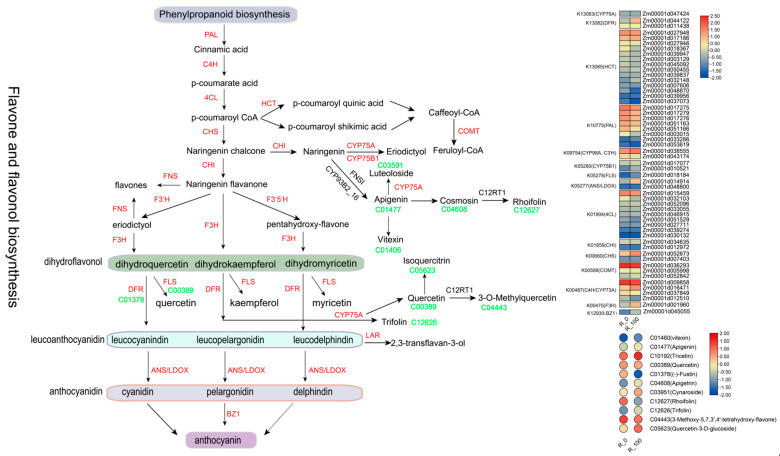
Flavonoid biosynthesis pathway and analysis of differential gene expression and metabolite differences (The red ones in the figure represent the differentially expressed structural genes, and the green ones represent the differentially metabolite identifiers).

Integrated metabolomic and transcriptomic analyses of the flavonoid biosynthesis pathway involved in the formation of maize root colors (Figure 5 and Table S6) revealed that anthocyanin biosynthesis involved the phenylpropanoid biosynthesis and flavonoid biosynthesis pathways. Among the genes related to flavonoid synthesis, *Zm00001d001960 (F3H)*, *Zm00001d044122 (DFR)*, and *Zm00001d014914 (ANS/LDOX)* were significantly positively regulated, and *PAL*-related *genes* (*Zm00001d003015*, *Zm00001d051166*, *Zm00001d017276*, and *Zm00001d017279*) and *4CL* genes (*Zm00001d032103* and *Zm00001d051529*) were significantly negatively regulated under MeJA treatment. In addition, the crucial gene *Zm00001d045055 (BZ1)* in the anthocyanin biosynthetic pathway was significantly upregulated following MeJA induction. These genes collectively facilitated the accumulation of anthocyanin. Among the differentially regulated metabolites in the metabolic pathway, C01378 exhibited a significant decrease after MeJA treatment, whereas C00389 showed a significant increase.

Transcription factors are crucial genes that regulate structural genes. Therefore, we conducted a correlation analysis between the expression of transcription factors bHLH and MYB, and the metabolites and structural genes related to the flavonoid synthesis pathway (Figure S1). The results indicated that, among the bHLH transcription factors, *Zm00001d017804*, *Zm00001d000236*, *Zm00001d037336* and *Zm00001d043706* exhibited higher R values, suggesting a stronger correlation with structural genes (Table S7). In contrast, *Zm00001d018097*, *Zm00001d029963* and *Zm00001d039492* within the MYB group were more closely related to the structural genes involved in the anthocyanin synthesis pathway (Table S7).

**Figure 6:**
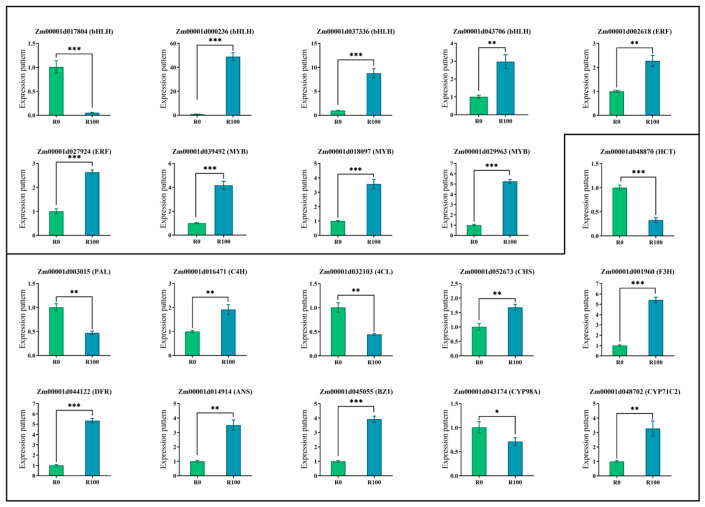
Analysis of differential expression patterns of key genes in the anthocyanin biosynthesis pathway and differential transcription factors (***: *p* value < 0.001; **: *p* value < 0.01; *: *p* value < 0.05).

Thorough analysis showed a total of 20 genes were ultimately selected for expression analysis, comprising anthocyanin biosynthetic structural genes and differentially expressed transcription factors. We first conducted screening of candidate reference genes. Utilizing gene expression data to assess variation, we evaluated five potential reference genes (*ACTIN1*, *ACTIN2*, *ACTIN11*, *EF-1α*, and *18S rRNA*). Following assessment of Ct values and expression stability in diverse samples, *EF-1α* was identified and chosen as the reference gene for all further investigations (Table S8). The results indicated that, overall, the expression patterns were consistent with the transcriptional data and exhibited significant differences (Figure 6 and Table S8). Notably, the expression levels of three transcription factors, namely *Zm00001d000236 (bHLH)*, *Zm00001d037336 (bHLH)*, and *Zm00001d043706 (bHLH)* were markedly different and all upregulated under MeJA induction. Additionally, the expression of the structural genes in Flavonoid synthesis pathway were all significantly enhanced. Except for *Zm00001d003015 (PAL)* and *Zm00001d032103 (4CL)*, in which expression was downregulated under MeJA induction, the remaining structural genes were upregulated.

## 4. Discussion

Jasmonate plays a crucial role in regulating the accumulation of anthocyanin and proanthocyanidin [30,31]. Anthocyanin and proanthocyanidin are plant secondary metabolites found in seeds, leaves, flowers and fruits [5]. In comparison to the control group, the growth and development of corn were relatively enhanced under MeJA treatment, and this study also yielded comparable results [32].

In this study, the plant seeds of a black corn inbred line with genetic stability were used, while other tissues such as stem segments exhibited the normal corn color. After MeJA induction treatment, a significant change in the color of the plant’s primary root system was observed, which is consistent with the findings of Leon-Cisneros et al., that MeJA induction of seeds leads to changes in the color of corn embryonic roots [33]. Based on this phenotype, we conducted analyses on gene transcriptional expression differences and metabolic variations, to elucidate the underlying mechanisms.

Through the analysis of transcriptomic data, differentially expressed genes were found to cluster within the flavonoid biosynthetic pathway. The flavonoid biosynthetic pathway plays a crucial role in both plant stress resistance and anthocyanin synthesis. In this study, the differentially expressed genes were primarily clustered within the phenylalanine and flavonoid-related regulatory networks, which is consistent with our research expectations. In the investigation of key structural genes related to the anthocyanin biosynthetic pathway, it was found that MeJA treatment significantly upregulated the expression of multiple structural genes involved in anthocyanin biosynthesis [34,35]. In our study, MeJA treatment significantly upregulated the expression of structural genes involved in the anthocyanin biosynthetic pathway, including *F3H* (flavanone 3-hydroxylase), *DFR* (dihydroflavonol 4-reductase), *ANS* (anthocyanidin synthase), and *BZ1* (UDP-glucose:flavonoid 3-O-glucosyltransferase, *UFGT*) (Figure 4 and Figure 5). These genes play key roles in the synthesis of anthocyanins, and their increased expression provides the foundation for the substantial biosynthesis of anthocyanins [36]. Consequently, we selected 20 genes with significant expression differences following induction and related to anthocyanin for expression analysis. Ultimately, we identified that the key regulatory genes in anthocyanin accumulation in maize roots under MeJA induction are likely *Zm00001d001960 (F3H)*, *Zm00001d014914 (ANS/LDOX)*, *Zm00001d044122 (DFR)* and *Zm00001d045055 (BZ1)* (Figure 5). And these genes were all significantly expressed in maize primer roots (https://www.maizegdb.org, (accessed on 3 August 2025)). This finding is similar to the results of Wang et al., in which using Prohydrojasmon could promote the expression of *ANS*, *F3H*, *DFR* and *UFGT* [37]. In addition, eggplant transcription factor SmMYB5 integrates jasmonate during anthocyanin biosynthesis, which regulates *F3H*, *DFR* and *ANS* [38].

Transcription factors can also effectively regulate structural genes, thereby influencing the accumulation of anthocyanins. While the top five differentially expressed TF families mediated JA-dependent stress/developmental responses [39,40,41,42,43], they showed negligible association with anthocyanin metabolism. Conversely, bHLH and MYB/MYB-related TFs, representing the most abundant DEG clusters, directly governed anthocyanin biosynthetic genes via promoter binding. The R2R3-MYB transcription factor is the main MYB transcription activator in the MBW (MYB-bHLH-WD40) protein complex responsible for regulating anthocyanin biosynthesis, and the expression levels of R2R3-MYB transcription factors in various plants are highly positively correlated with anthocyanin content [44]. We conducted preliminary sequence analysis on bHLH and MYB transcription factors identified as exhibiting significant correlations with structural genes. Ultimately, after filtering out extremely short sequences and conducting NCBI-CDD analysis, we identified 31 bHLH and 13 MYB genes containing transcription factor-specific domains.

The qPCR results revealed that the bHLH genes (*Zm00001d017804*, *Zm00001d000236*, and *Zm00001d037336*) exhibited highly significant differences in expression. But the Conserved Domain Database analyzed only *Zm00001d000236* and *Zm00001d017804* with bHLH domain information. This indicates that bHLH transcription factors in maize play a crucial regulatory role in responding to induction and altering plant color. The study by Liu et al. demonstrated that SmbHLH60 negatively regulates anthocyanin expression, and, under MeJA mediation, it antagonistically regulates phenolic acid and anthocyanin biosynthesis with *SmMYC2* [45]. For instance, the bHLH transcription factor *MdbHLH162* has been shown to integrate gibberellin (GA) and jasmonic acid (JA) signals, to negatively regulate anthocyanin biosynthesis. This regulatory mechanism is consistent with findings that bHLH transcription factors often play crucial roles in the biosynthesis of secondary metabolites, including anthocyanins [46]. bHLH42 was robustly induced by MeJA, and closely correlates with tissue-specific accumulation of anthocyanins in *Caitai* [47]. Correlation analysis also revealed that the bHLH gene *Zm00001d000236* exhibited high correlation with *Zm00001d044122 (DFR)* and *Zm00001d001960 (F3H)*. These genes were significantly upregulated under high concentrations of MeJA stress, thereby promoting anthocyanin biosynthesis [48].

In addition to bHLH transcription factors, MYB transcription factors also played a crucial role in regulating anthocyanin biosynthesis. In this study, the MYB transcription factor gene *Zm00001d018097* exhibited significant differences in expression before and after induction (Figure 5). MYB transcription factors are well-known regulators of anthocyanin biosynthesis. For instance, in apple, MYB transcription factors have been identified as key regulators of anthocyanin biosynthesis, contributing to the red flesh color [37,49]. The PcMYB10-PcMYC2 molecular complex has been proposed to regulate flavonoid biosynthesis at the transcriptional level in a JA-mediated manner [50]. Correlation analysis of MYB transcription factors, flavonoid-related genes, and metabolites in this study revealed that *Zm00001d018097* and *Zm00001d029963* were highly correlated with *Zm00001d044122 (DFR)*, *Zm00001d001960 (F3H)*, *Zm00001d014914(ANS/LDOX)* and *Zm00001d045055 (BZ1)*. Therefore, it can be inferred that the MYB gene promotes anthocyanin biosynthesis by regulating the expression of structural genes.

To further investigate whether MYB and bHLH transcription factors could regulate the expression of these four genes, we predicted the promoter regions of these structural genes. Within the 2000 bp region upstream from the start codon, we conducted comparative analysis of 22 bHLH and MYB binding motifs. This analysis revealed that the promoters of F3H, ANS, Zm00001d045055, and Zm00001d029963 (MYB) harbor bHLH binding sites (https://jaspar.elixir.no/matrix/MA1834.2/ (accessed on 16 August 2025)). Similarly, MYB binding sites were found in the promoter regions of DFR, ANS, BZ1, and Zm00001d000236 (bHLH) (https://jaspar.elixir.no/matrix/MA1829.2/ (accessed on 16 August 2025)) (Table S10).

Therefore, we integrated significant co-expression relationships between structural genes and bHLH/MYB transcription factors (Table S7), statistical interactions between these regulators, and differential expression patterns, to elucidate the regulatory network. Therefore, we inferred that MeJA induction in maize roots promotes the expression of *Zm00001d018097 (MYB)*, *Zm00001d029963 (MYB)* and *Zm00001d000236 (bHLH)*. The combined action of these two transcription factors subsequently enhances the expression of the anthocyanin biosynthesis-related genes *F3H* and *DFR*, leading to anthocyanin accumulation [45,51]. The molecular mechanism involves two potential pathways: on the one hand, the bHLH-MYB complex may facilitate *DFR* expression through direct binding of the MYB subunit to its promoter region. On the other hand, the same complex might promote *F3H* expression via direct interaction of the bHLH subunit with its cognate promoter.

## 5. Conclusions

In this investigation, we elucidated the impact of MeJA on anthocyanin accumulation within maize root systems, revealing not only significant alterations in anthocyanin content upon MeJA elicitation, but also demonstrating that stress resilience conferred by root-localized anthocyanins enhances overall plant development. Through the integrated analysis of transcriptomics and metabolomics, we indicated a substantial number of differentially expressed genes and metabolites that clustered within the flavonoid biosynthesis pathway. These findings strongly indicate that MeJA-induced anthocyanin accumulation predominantly impacts flavonoid biosynthesis, with a particular emphasis on modulating the expression of the *F3H* and *DFR* genes. Regarding transcription factors, our analysis revealed significant enrichment in two major families: bHLH and MYB. Expression pattern analysis further elucidated the critical regulatory roles of specific genes during the induction process.

Based on these comprehensive findings, we propose a regulatory model in which bHLH and MYB transcription factors (*Zm00001d018097 (MYB)*, *Zm00001d029963 (MYB)* and *Zm00001d000236 (bHLH)*) act synergistically to promote the expression of *F3H* and *DFR*, thereby amplifying MeJA-elicited anthocyanin biogenesis in maize roots and fortifying subterranean stress-defense competencies. This model not only deepens our understanding of the molecular mechanisms underlying phytohormone-regulated anthocyanin biosynthesis, but also provides valuable genetic targets for future breeding strategies aimed at enhancing the nutritional quality and stress resistance of maize.

## Data Availability

Data are provided within the manuscript or Supplementary Information Files.

## Supplementary Materials

The following supporting information can be downloaded at: https://www.mdpi.com/article/10.3390/biology14091124/s1, Figure S1: Analysis between the transcription factors bHLH/ERF and MYB, and the metabolites and structural genes related to the flavonoid synthesis pathway; Table S1: Differential analysis of maize development and anthocyanin content after MeJA treatment. Phenotype: phenotypic trait measurements; Phenotype-anova: statistical significance analysis for phenotypic variation; Anthocyanidin: analytical measurements of anthocyanidin compounds; Anthocyanidin-anova: significance analysis of anthocyanidin accumulation; Table S2: Quality control data of transcriptome sequencing samples; Table S3: (1) Correlation plot among samples: intrasample differential analysis of transcriptome samples; (2) DEG_significant: significantly differentially expressed genes; (3) top30_GO: GO annotation in DEGs; (4) KEGG_pathway_enrichment: KEGG enrichment in DEGs; (5) Count of TFs: statistics of differential transcription factors; (6) map00941_gene: differentially expressed genes related to the flavonoid biosynthesis pathway; Table S4 (1) Merge rate: distribution of metabolites in untargeted metabolomics; (2) DEM_sigificant: significantly differential metabolites; (3) DEM_PATHWAY: their KEGG enrichment analysis; (4) Meta_flavonoid pathway: differentially metabolites related to the flavonoid biosynthesis pathway; Table S5: Correlation analysis between differentially expressed genes and metabolites in the flavonoid pathway; Table S6: Analysis of differences in the expression of differentially expressed genes (Gene_expression) and metabolite contents in the flavonoid pathway (Meta_content); Table S7: Analysis of the correlation between differentially expressed genes and metabolites in the flavonoid pathway and transcription factors bHLH and MYB; Table S8: qPCR analysis of target gene expression levels. qPCR_primers: primer pair specifications; reference_results: Ct values of validated reference genes; qPCR_results: relative quantification (2^−ΔΔCt^ method); ANOVA: statistical analysis of expression variance; Table S9: Correlation analysis of bHLH and MYB transcription factors; Table S10: The number of bHLH and MYB binding sites in structural genes’ promoter; Table S11: Hogland nutrient solution formula.
