# Supplementary figures and images for: Integrated Transcriptomic and Metabolomic Analysis of Color Changes in Maize Root Systems Treated with Methyl Jasmonate
Source: Biology (Basel). 2025 Aug 25;14(9):1124. doi: 10.3390/biology14091124 (PMC12467196; doi:10.3390/biology14091124)

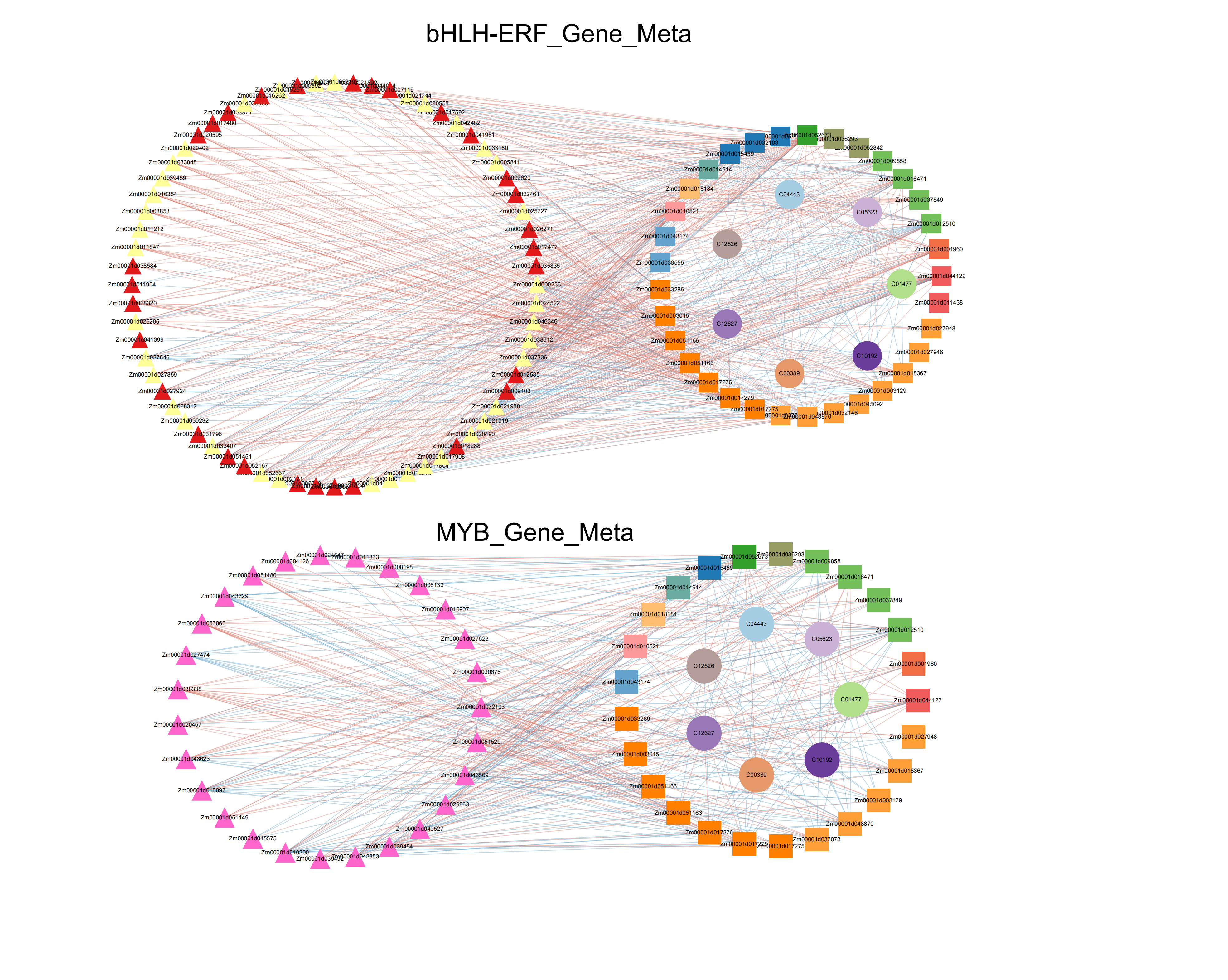

Supplement: Supplementary file 1 [file biology-14-01124-s001.zip › Figure S1.png]
